# The Oatmeal Cure in Diabetes

**Published:** 1907-03

**Authors:** 


### THE OATMEAL CURE IN DIABETES.

The Von Noorden oatmeal cure consists of 250 gm. of oatmeal, which is cooked with 300 gm. of butter and water into a soup, to which 100 gm. of Roborat, consisting of 80 per cent. albumin, are added. This is fed to the patients in the form of soup every two or three hours. The patients, therefore, obtain 2,500 calories in the form of fat, and about 400 calories in the form of albumin, which is sufficient to protect the body albumin, and adequate for a diabetic weighing 80 kg., even though he excretes 50 to 100 gm. sugar a day. Diabetic patients put upon this diet, therefore, receive, in addition to a perfectly adequate diet, a large amount of carborydrate. Experience shows that severe diabetics are unable to assimilate carbohydrates, but excrete the same in the urine, while temporarily with the increase of glycosuria the acidosis may fall. Since Von Noorden's conception would lead to the conclusion that the starch of oatmeal acts specifically otherwise in the metabolism than all other hexoses, before accepting this hypothesis Lipetz believed sources of error should be sought.

One possibility is that patients do not absorb the carbohydrates at all, or very badly, and simply live from the fat and albumin. Lipetz therefore studied the dried stools of patients on ordinary hospital diet and on Von Noorden's oatmeal cure, but they showed no lack of absorption. Another possibility is that increased fermentation takes place in the intestine, leaving no sugar as such to be absorbed. Two explanations are, therefore, to be considered: either the carbohydrates are in the most part quickly absorbed, as a result of which sugar will appear in the urine, with no increase in the amount of fermentation as shown by the bacteria in the feces, or there will occur a very marked increase in fermentation with increased amounts of fecal bacteria, but no increase of glycosuria. Lipetz studied this question first on a patient convalescent from rheumatism, but without diabetes. He found that the amount of bacteria increased in the period of the oatmeal diet from 9.82 gm. up to 14.57 gm. In a second experiment on the same person the increase of bacteria was about 40 per cent., while the weight fell from 55.1 kg. to 54.4 kg. The oatmeal diet was then given to a severe case of diabetes. As a result the quantity of sugar excreted markedly increased during the oatmeal period as compared with preceding and following periods. As expected, no increase took place in the bacteria of the stools. The patient died a few days later from coma, but this was probably not related to the diet, but rather to the fact that bicarbonate of soda was not given in the usual quantities. In another case of diabetes of moderate severity the patient on the oatmeal diet showed an increase of only 10 gm. of carbohydrates, which would suggest that the carbohydrates of the oatmeal were only to the slightest part absorbed, but in great part fermented. This view was confirmed by the marked increase in the amount of bacteria in the stools. Before the oatmeal diet was given, the total quantity of sugar in the urine had fallen from 14.4 to 7 gm. During the oatmeal period the urine became almost entirely sugar free, but the sugar reappeared as soon as there was a return to the ordinary diet. The oatmeal diet was given to the patient, but this time the excretion of sugar increased. Several other patients either vomited after taking the oatmeal diet for some days, or refused it. The good results which others appear to have obtained with the oatmeal cure appear, therefore, to be due to the fact that the carbohydrates are little, or not at all, absorbed. Whether the products of fermentation which were absorbed exercise any specific action is unknown.—Medical Standard, February, 1907.

